# Monitoring of noninvasive ventilation: comparative analysis of different strategies

**DOI:** 10.1186/s12931-020-01586-8

**Published:** 2020-12-10

**Authors:** Marjolaine Georges, Claudio Rabec, Elise Monin, Serge Aho, Guillaume Beltramo, Jean-Paul Janssens, Philippe Bonniaud

**Affiliations:** 1 Department of Pulmonary Medicine and Intensive Care Unit, University Hospital, Dijon, France; 2 University Burgundy Franche-Comté, Dijon, France; 3 Centre Des Sciences du Goût Et de L’Alimentation, UMR 6265 CNRS 1234 INRA, University Burgundy Franche-Comté, Dijon, France; 4 Department of Epidemiology, Dijon University Hospital, Dijon, France; 5 Division of Pulmonary Diseases, Geneva University Hospitals, Geneva, Switzerland; 6 INSERM U1231, University Burgundy Franche-Comté, Dijon, France; 7 Service de Pneumologie Et Soins Intensifs Respiratoires, Centre Hospitalier Et Universitaire de Dijon, Hôpital François Mitterrand, 14 rue Paul Gaffarel, 21079 Dijon, France

**Keywords:** Non-invasive ventilation, Monitoring, Transcutaneous capnography, Nocturnal pulse oximetry, Bi-level positive airway pressure, Respiratory failure

## Abstract

**Background:**

Noninvasive ventilation (NIV) represents an effective treatment for chronic respiratory failure. However, empirically determined NIV settings may not achieve optimal ventilatory support. Therefore, the efficacy of NIV should be systematically monitored. The minimal recommended monitoring strategy includes clinical assessment, arterial blood gases (ABG) and nocturnal transcutaneous pulsed oxygen saturation (SpO_2_). Polysomnography is a theoretical gold standard but is not routinely available in many centers. Simple tools such as transcutaneous capnography (TcPCO_2_) or ventilator built-in software provide reliable informations but their role in NIV monitoring has yet to be defined. The aim of our work was to compare the accuracy of different combinations of tests to assess NIV efficacy.

**Methods:**

This retrospective comparative study evaluated the efficacy of NIV in consecutive patients through four strategies (A, B, C and D) using four different tools in various combinations. These tools included morning ABG, nocturnal SpO_2_, TcPCO_2_ and data provided by built-in software via a dedicated module. Strategy A (ABG + nocturnal SpO_2_), B (nocturnal SpO_2_ + TcPCO_2_) and C (TcPCO_2_ + builtin software) were compared to strategy D, which combined all four tools (NIV was appropriate if all four tools were normal).

**Results:**

NIV was appropriate in only 29 of the 100 included patients. Strategy A considered 53 patients as appropriately ventilated. Strategy B considered 48 patients as appropriately ventilated. Strategy C misclassified only 6 patients with daytime hypercapnia.

**Conclusion:**

Monitoring ABG and nocturnal SpO_2_ is not enough to assess NIV efficacy. Combining data from ventilator built-in software and TcPCO_2_ seems to represent the best strategy to detect poor NIV efficacy.

*Trial* *registration* Institutional Review Board of the Société de Pneumologie de Langue Française (CEPRO 2016 Georges)

## Background

Non-invasive ventilation (NIV) is recognized as an effective treatment of chronic hypercapnic respiratory failure (CHRF) [1]. Due to growing evidence of NIV efficacy in a broad range of indications as well as increasing availability of high performance and user-friendly home ventilators, the number of patients receiving NIV at home has been regularly increasing over the past 30 years [2–4]. When NIV is initiated to treat CHRF, ventilator settings are empirically determined based on the underlying disease, patient tolerance and diurnal changes in arterial blood gases (ABG) [5]. However, NIV is usually applied during the night. As a result, daytime adjustment of ventilator settings may not achieve optimal nocturnal ventilatory support. This can be explained by sleep-related changes in breathing. Sleep induces modifications in ventilatory control, respiratory muscle recruitment and upper airway patency, which may all affect ventilatory function especially in patients with CHRF [6]. Moreover, applying intermittent positive pressure may by itself trigger abnormal respiratory events [7]. For instance, reduction of ventilatory drive with or without glottic closure, residual upper airway obstruction and patient-ventilator asynchrony can all compromise the efficacy of NIV [7]. Furthermore, as NIV uses a non-airtight system, unintentional leaks are frequent [8]. Leaks during NIV can interfere with patient-ventilator interaction [9]. These respiratory events are frequent under NIV [8, 10–13] and may have an impact on prognosis [14–16].

Therefore, NIV should be systematically monitored. However, optimal modalities for monitoring of long-term ventilated patients remain a matter of debate. Hence, physicians may adopt different approaches to assess NIV performance. Some authors suggest that complete polysomnography (PSG) under NIV should be performed for each patient under NIV to verify its efficacy [7, 17]. This technique is not feasible in many centres on a routine basis. In contrast, the 2010 American Academy Sleep Medicine (AASM) recommendations for best clinical practices state that patients on long term NIV should be assessed regularly with measures of oxygenation and ventilation (i.e.: ABG, nocturnal pulse oximetry, end tidal CO_2_ or transcutaneous capnography) [18, 19]. Over the past years, the use of TcPCO_2_ has been simplified. Home ventilators have built-in software that provide detailed information on relevant ventilator parameters to assess the efficacy of NIV. A step-by-step strategy starting by ABG and nocturnal SpO_2_ has been proposed by the SomnoNIV group [19]. However, few studies have evaluated these proposed monitoring strategies in clinical practice [20].

This study aimed to compare the accuracy of four different strategies using four easily available assessment tools in different combinations to determine NIV efficacy during elective evaluations of patients on long-term NIV.

## Methods

All patients under long-term home NIV followed by the Pulmonary Department of Dijon University Hospital are hospitalized electively for one night on a regular basis to assess efficacy of their NIV. These admissions are scheduled by the attending specialist every 3 to 12 months: intervals depend on the underlying respiratory disease and its progression rate, prior assessment of NIV efficacy or tolerance and intercurrent medical events.

In this retrospective comparative study, we included consecutive patients treated with long term NIV and hospitalized in our unit for an elective follow-up visit over a 1 year period. Inclusion criteria were: use of a home bi-level pressure support ventilator (VPAP™, ResMed, North Ryde, Australia) and being in a stable clinical condition for at least 3 months prior to inclusion.

Exclusion criteria included: age below 18 years, oxygen supplementation, use of a ventilator from other manufacturers, mean daily NIV use of less than 4 h per night, inability to cooperate and change in NIV treatment in the preceding 3 months.

NIV was evaluated with usual ventilator settings and interface. We simultaneously recorded overnight for each patient four monitoring tools: (1) morning ABG measured during spontaneous breathing by puncture of the radial artery during the first hour after disconnection from the ventilator, (2) nocturnal pulsed oxygen saturation (SpO_2_; Nonin model 8500 oximeter, Nonin Medical, Plymouth, MN, USA), (3) transcutaneous capnography (TcPCO_2_: Tosca^®^, Radiometer, Copenhagen, Denmark) and (4) data from a simplified monitoring module coupled to their portable ventilator (Reslink™, ResMed). Data from the ventilator software were collected on a Smart Media card (Scandisk, Milpita, CA, USA) then downloaded with Rescan™ software (ResMed, North Ryde, Australia). The software provided an accurate estimation of non-intentional air leaks (i.e. leaks exceeding what was expected from the exhalation valve of the interface used) [8]. The additional connection of a pulse oximeter allowed simultaneous recording of nocturnal SpO_2_.

Thresholds used to interpret results of the four monitoring tools were the following: (1) ABG: PaCO_2_ ≥ 45 mmHg; (2) nocturnal SpO_2_: time spent with SpO_2_ < 90% for ≥ 30% of the total recording time [21]; (3) transcutaneous capnography: mean TcPCO_2_ ≥ 50 mmHg [22, 23] and (4) data from built-in ventilator software: leaks (> 24 l/min for > 20% of total recording time), continuous desaturation (SpO_2_ < 90% for > 30% of the recording) and cumulated desaturation dips (> 3% during > 10% of the trace) [8].

We evaluated the efficacy of NIV through four strategies (A, B, C and D) using the results of four different tools, in different combinations: *strategy A* combined ABG and nocturnal SpO_2_, the minimal recommended monitoring combination [19]; *strategy B* combined nocturnal SpO_2_ and TcPCO_2_: since transcutaneous capnography provides SpO_2_ and TcPCO_2_ simultaneously, both parameters could be analyzed concurrently; *strategy C* combined TcPCO_2_ and data from built-in ventilator software and *strategy D* associated all the available tools (i.e. ABG, nocturnal SpO_2,_ TcPCO_2_ and data from ventilator software). *Strategy D* is used to classify patients as appropriately ventilated or not. If none of the above-mentioned criteria were fulfilled, NIV was considered effective.

The St. Mary’s Hospital questionnaire was completed in the morning after the overnight assessment to evaluate subjective sleep quality on a 12 point scale [24]. Another questionnaire assessed the self-perceived quality of ventilation using an eight-item visual analogic scale (10 points per item) covering three domains: patient-ventilator synchronisation, efficacy and leaks [25]. Higher values indicated better treatment comfort, with a maximum score of 80.

The study was approved by the Institutional Review Board of the Société de Pneumologie de Langue Française.

### Statistical analysis

Statistical analyses were performed using SigmaPlot 13 software (Systat Software, San Jose, CA, USA). The normality of the distribution of the variables analysed was assessed using the Kolmogorov–Smirnov test. As most data were not normally distributed, we reported results as median and quartiles and used non-parametric tests. We used the Mann Whitney’s U test to compare “appropriately” and “inappropriately” ventilated patients for continuous variables. Categorial variables (gender, interfaces) were compared using a χ^2^ test. For comparisons between three or more groups (classification of patients according to the aetiology of chronic respiratory failure), we used the Kruskal–Wallis test; subsequent paired comparisons were made using a post-hoc Dunn’s analysis. Statistical significance was set at p < 0.05 or p < 1 − (1 − α)^1/k^ for multiple comparisons where α = 0.05 and k denotes the number of comparisons.

The agreement between different methods of NIV monitoring and the *strategy D* was evaluated with Cohen’s kappa coefficient [26].

We used receiver operating characteristic (ROC) curves to evaluate the performance of nocturnal SpO_2_ and ABG to identify patients classified as adequately ventilated according to *strategy D*. We considered agreement to be sufficient if the lower bound of 95% confidence interval for the area under the ROC curve was > 0.7. ROC curve analyses were also used to determine the most suitable threshold values of mean nocturnal SpO_2_ and morning PaCO_2_ for assessing NIV efficacy.

## Results

One hundred and thirty-four patients were screened. Two subjects were excluded due to corruption of raw data from the ventilator software. Thirty-two patients under oxygen therapy were also excluded from further analyses. These subjects suffered more often from obstructive lung diseases (OLD) and presented more severe diurnal and nocturnal hypercapnia (p < 0.001).

### Study population

The remaining 100 patients were treated with NIV for OLD (n = 25), chest wall diseases (CWD, n = 29) and neuromuscular diseases (NMD, n = 46) according to the Eurovent diagnostic groups [2] (Table 1). Demographic characteristics, ABG, TcPCO_2_ and ventilator settings are summarized in Table 2. As expected, NMD patients were younger, had a lower BMI and required lower levels of pressure support to reach more effective control of diurnal and nocturnal hypercapnia. Nasal masks were used more frequently in this group than in OLD or CWD subjects (p < 0.05).

**Table 1:** Characteristics of the studied population: indications for noninvasive ventilation according to Eurovent categories

Aetiologic group	Subjects
Obstructive lung diseases (OLD)	25
Chronic obstructive pulmonary disease	12
Overlap syndrome	10
Other	3
Chest wall diseases (CWD)	29
Obesity hypoventilation syndrome	21
Tuberculosis sequelae	3
Kyphoscoliosis	5
Neuromuscular diseases (NMD)	46
Myopathy	25
Amyotrophic lateral sclerosis	13
Neuropathy	8

Data are presented as number of subjects

**Table 2:** Characteristics of the studied population: demographic data, diurnal and nocturnal gas exchanges and ventilator settings

Variables	OLD	CWD	NMD	Global population
Effective	25	29	46	100
Anthropometric data				
Age (years)	70 [61–77]	71 [60–75]	50 [22–62]*^¶^	62 [47–71]
Gender (male/female)	16/9	12/17	31/15	59/41
BMI (kg/m^2^)	39 [32.2–41.5]	40 [27.2–47.7]	22 [18.4–30.5]*	31.0 [21.6–40.4]
Daytime arterial blood gases				
Daytime PaO_2_ (mmHg)	66.5 [61.1–79.3]	65 [60.7–73]	78 [71–93]*^¶^	71.3 [62.4–84]
Daytime PaCO_2_ (mmHg)	45 [41.9–49]	42 [39.3–47.8]	41 [38.5–44.2]*	41.9 [39.4–47]
Nocturnal transcutaneous capnography				
Median SpO_2_ (%)	90 [89–92.4]	92 [89.8–93.3]	95 [93.8–96]*	93 [90–95]
Median TcPCO_2_ (mmHg)	49 [44.2–53.2]	48 [43–52.4]	43 [40–47.3]*^¶^	45.8 [41.9–50.1]
Maximal TcPCO_2_ (mmHg)	55 [49.5–64]	56 [52.5–59]	48 [44–53.5]*	52 [46–57]
Recording time spent with TcPCO_2_ > 50 mmHg	58 [1.96–89.9]	28 [3.3–81.5]	0 [0–28.2]*	6.7 [0–72.1]
Ventilator settings				
Inspiratory pressure (cmH_2_O)	19 [18–21]	18 [16–19]	16 [14–17]*^¶^	17.5 [16–19]
Expiratory pressure (cmH_2_O)	8 [6–9]	9 [5–10]	6 [4–8]*^¶^	6 [4–10]
Interface: nasal/oronasal mask	12/13	13/16	28/18*^¶^	53/47
Compliance (h/day)	8.5 [7.5–10]	7.2 [5.8–9.7]	8 [6–9.4]	7.9 [6.1–9]

Data are presented as median [first and third quartiles] or number of subjects

*BMI* body mass index, *CWD* chest wall diseases, *NIV* noninvasive ventilation, *NMD* neuromuscular diseases, *OLD* obstructive lung diseases, *PaCO*_*2*_ arterial carbon dioxide partial pressure, *PaO*_*2*_ arterial dioxygen partial pressure *SpO*_*2*_ transcutaneous pulsed oxygen saturation, *TcPCO*_*2*_ transcutaneous carbon dioxide partial pressure

^*^ p < 0.05 for comparisons to OLD group

^¶^ p < 0.05 for comparisons to CWD group (Kruskall–Wallis test then Dunn’s post-hoc analysis or χ^2^)

### Assessment of NIV efficacy

TcPCO_2_ revealed significant nocturnal hypoventilation in 27% of the patients. Among them, 6% had normal ABG and 12% had normal nocturnal SpO_2_. Data from built-in ventilator software were abnormal in 57% of the patients. Leaks represented the most common abnormality (28%).

Table 3 compares the performances of different strategies. NIV was appropriate in only 29% of patients. No significant differences were found regarding ventilator settings or interfaces between appropriately and inappropriately ventilated patients. NIV compliance did not differ significantly between appropriately and inappropriately ventilated patients (8.5 [6.9–10] vs. 7.5 [6.1–9.9] hours per night, respectively).

**Table 3:** Proportion of patients considered as appropriately ventilated according to tests used alone or in various strategies

Evaluation criteria	Patients fulfilling criteria for appropriate ventilation according to tests performed alone or in combination	Cohen’s к coefficient^a^
Assessment tools used alone		
Data from Bbuilt-in ventilator software polygraphy	43 (43%)	0.685 [0.545–0.825]
TcPCO_2_	73 (73%)	0.332 [0.201–0.465]
Assessment tools used in combination		
ABG + nocturnal SpO_2_ (*strategy A*)	53 (53%)	0.557 [0.406–0.707]
Nocturnal SpO_2_ + TcPCO_2_ (*strategy B*)	48 (48%)	0.601 [0.436–0.755]
Data from Bbuilt-in ventilator software polygraphy + TcPCO_2_ (*strategy C*)	35 (35%)	0.943 [0.876–1]
ABG + nocturnal SpO_2_ + ventilator softwareBuilt-in polygraphy + TcPCO_2_ (*strategy D*)	29 (29%)	

Data are presented as n (%)

Abnormal arterial blood gases defined as: PaCO_2_ ≥ 45 mmHg

Abnormal nocturnal SpO_2_ defined as: time spent with SpO_2_ < 90% for ≥ 30% of total recording time [21]

Abnormal nocturnal TcPCO_2_ defined as: mean TcPCO_2_ ≥ 50 mmHg [22, 23]

Abnormal data from built-in ventilator software polygraphy defined as abnormal if: 1/leaks (> 24 l/min for > 20% of total recording time); 2/continuous desaturation (SpO_2_ < 90% for > 30% of recording) or 3/cumulated desaturations (> 3% during > 10% of recording) [8]

*ABG* arterial blood gases; *SpO*_*2*_ transcutaneous pulsed oxygen saturation, *TcPCO*_*2*_ transcutaneous carbon dioxide partial pressure

^a^ The capacity of different methods of NIV monitoring was evaluated with Cohen’s к coefficient in comparison to *strategy D*

*With strategy A*, 53% of patients were considered appropriately ventilated. Among 48% of patients with normal results using *strategy B*, data from built-in ventilator software identified major leaks in 18% and significant drops in SpO_2_ associated with decreases in flow despite effective ventilator pressure in 10% of patients.

When using *strategy C*, NIV was considered appropriate in 35% of patients. Among them, only 6% had abnormal ABG and were misclassified. *Strategy C* performed better than *strategies A or B* for classifying appropriately vs. inappropriately ventilated patients (Cohen’s kappa coefficient, к for *strategy A *vs.* D*: 0.56 [0.41–0.71]; *strategy B* vs. *D*: 0.601 [0.436–0.755]; *strategy C* vs. *D*: 0.94 [0.86–1]).

### *Optimal threshold values for PaCO*_*2*_* and SpO*_*2*_* for identifying suboptimal NIV according to strategy D*

Table 4 presents ROC curve analysis of optimal threshold value of ABG and nocturnal SpO_2_ for identifying appropriately ventilated patients (defined by *strategy D*).

**Table 4:** Results of receiver operating characteristic curve analyses for mean nocturnal SpO_2_, time spent with SpO_2_ < 90% and morning PaCO_2_ for the detection of inappropriate NIV (according to *strategy D*)

	Threshold	Sensitivity (%)	Specificity (%)	Positive likelihood ratio	Negative likelihood ratio
Mean nocturnal SpO_2_ (%)	88	7.1	100	1.08	1
	90	32.4	100	4.38	0.69
	92	45.9	91.7	5.51	0.59
	94	71.6	70.8	2.46	0.40
	96	90.5	29.2	1.28	0.32
Time spent with SpO_2_ < 90% (% of total recording time)	5	63.5	95.8	15.24	0.38
	20	43.2	95.8	10.38	0.59
	30	43.2	100		0.57
Morning PaCO_2_ (mmHg)	42	69.9	90.9	7.68	0.33
	45	50.7	95.4	14.16	0.37
	48	36.2	100		0.64

*PaCO*_*2*_ arterial carbon dioxide partial pressure, *SpO*_*2*_ transcutaneous pulsed oxygen saturation

**Fig. 1:**
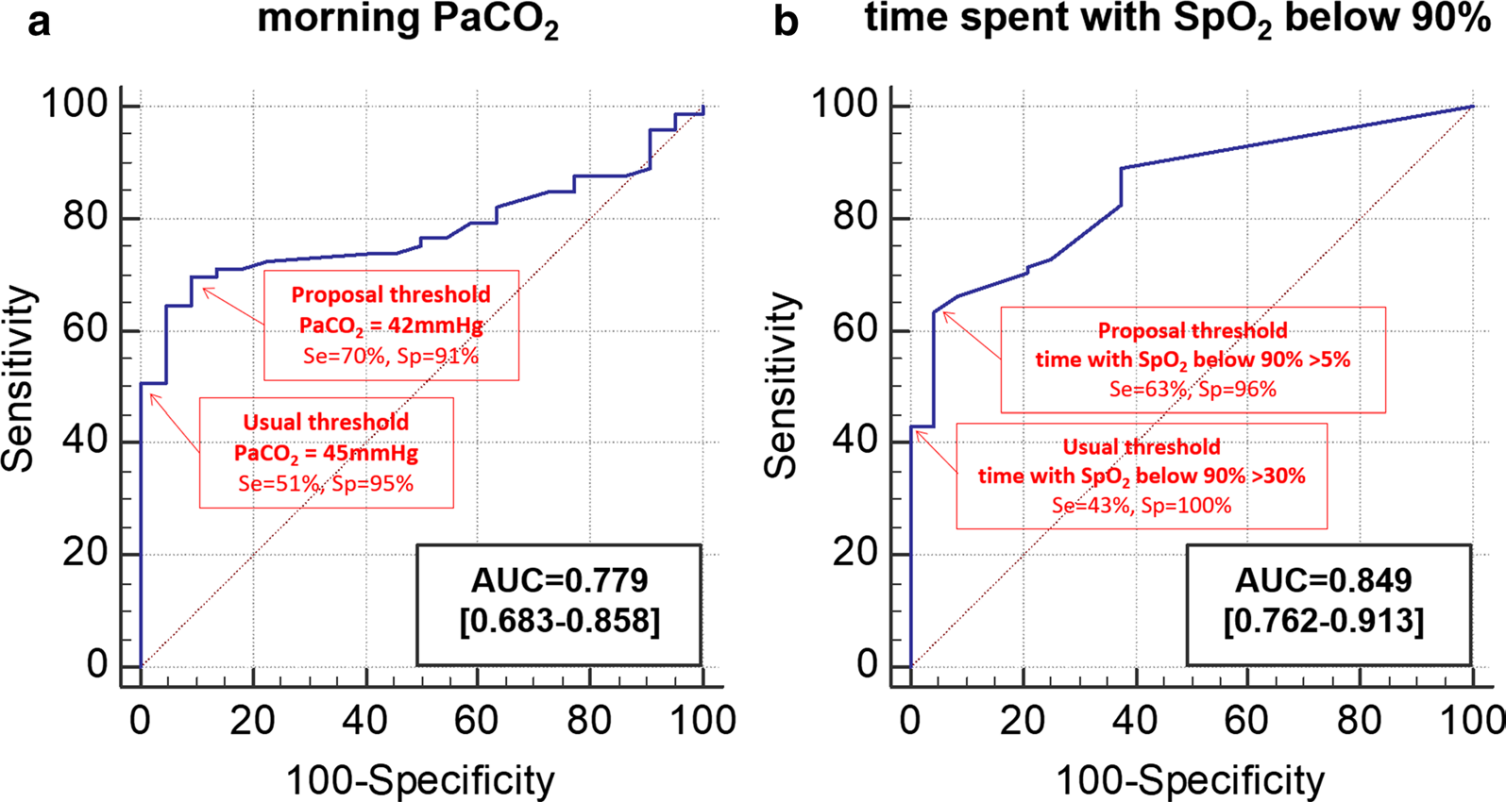
ROC curve of morning PaCO_2_ (**a**) and time spent with SpO_2_ below 90% (**b**) predicting NIV efficacy established by strategy D

A morning PaCO_2_ value of 42 mmHg was the best threshold for identifying appropriate NIV (Fig. 1a): 69% of the patients were correctly classified using this value.

The best threshold for time spent with SpO_2_ below 90% was 5% (Fig. 1b): 63% of the patients were correctly classified using this value. Higher values for time spent with SpO_2_ below 90% had a lower sensitivity with a similar specificity.

### Subjective assessment of quality of sleep and comfort of ventilation

**Fig. 2:**
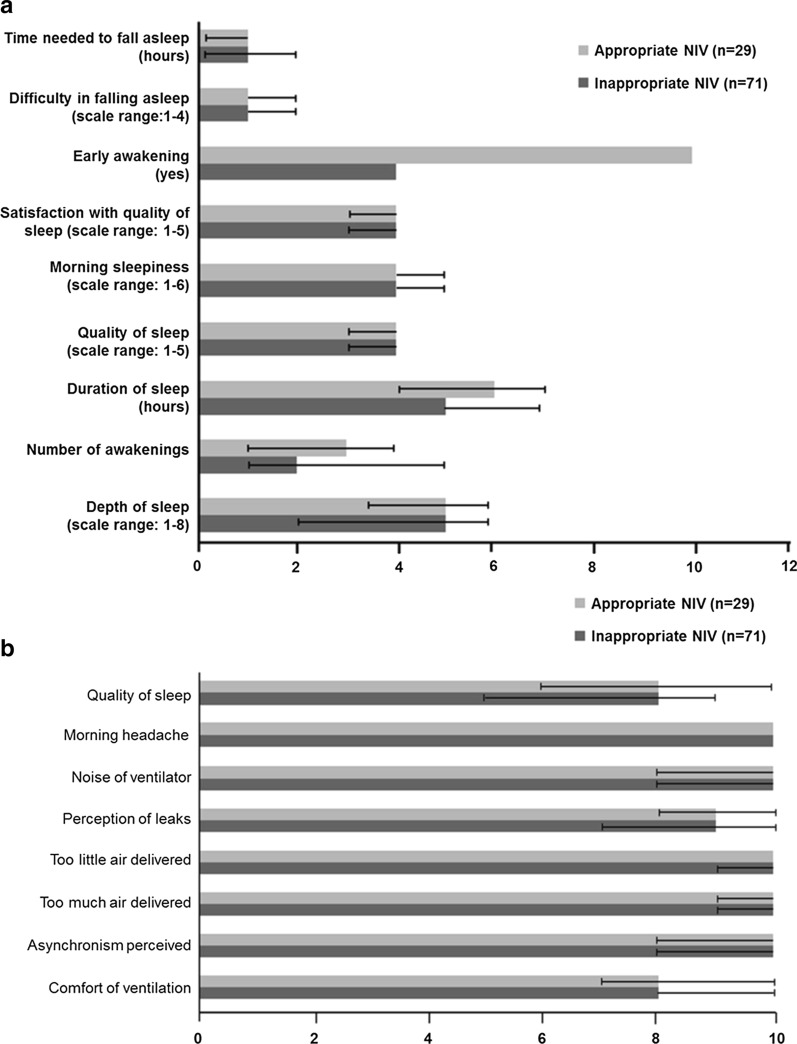
Patient’s rating of quality of sleep and ventilation assessed by St. Mary’s Hospital Questionnaire (**a**) and eight visual analogic scales (**b**) according to objective efficacy of NIV

Perceive quality of sleep (Fig. 2a) and comfort of ventilation (Fig. 2b) did not differ significantly between appropriately and inappropriately ventilated patients. Neuromuscular patients reported a worse quality of sleep and increased fragmentation (see Additional file 1 for perceived sleep quality and comfort of ventilation according to Eurovent categories).

## Discussion

In this real-life study, we compared different strategies to assess the efficacy of NIV. Our results suggest that using a combination of daytime ABG and nocturnal SpO_2_ (referred to as *strategy A*, proposed by the group of experts [19]) was not sensitive enough to assess NIV efficacy. A significant part of this group had residual nocturnal abnormalities under NIV (hypoventilation, unintentional leaks or abnormal events). In this group, withholding from performing further NIV testing could be deleterious. A combination of TcPCO_2_ and data from ventilator software, referred to as *strategy C*, was the most accurate non-invasive strategy for assessing NIV efficacy.

Improving NIV efficacy is an important issue in patients with long-term NIV: residual respiratory events under NIV may have a negative impact on patient-related outcomes such as symptoms, health-related quality of life and survival. Nocturnal hypoventilation is associated with a decreased survival rate, especially in neuromuscular diseases [14, 16], as well as adverse neuro-cognitive and cardiovascular consequences in chronic respiratory failure [27]. Leaks above 0.4 l/s [28] may induce patient-ventilator asynchrony [12, 29], alter quality of sleep [30–33] and potentially decrease health-related quality of life. Abnormal respiratory events under NIV (upper airway obstructive events with or without nocturnal desaturations or residual hypoventilation or symptoms) are associated with a decreased survival rate in patients suffering from amyotrophic lateral sclerosis (ALS) [15].

To detect residual nocturnal hypoventilation, we suggest using TcPCO_2_ instead of morning ABG. In ventilated patients, PaCO_2_ measured by arterial puncture may not provide an accurate picture of the overnight time course of PaCO_2_ [19, 22]. Several studies have shown that continuous TcPCO_2_ recording is well correlated with arterial measurements in chronic respiratory failure under NIV [10, 34, 35].

Experts propose different thresholds to assess the efficacy of NIV but little evidence substantiates the relevance of these values. Regarding TcPCO_2_, several thresholds have been suggested to define significant nocturnal hypercapnia: maximal TcPCO_2_ > 49 mmHg [36, 37]; TcPCO_2_ > 49 mmHg for > 10% of recording time [22]; TcPCO_2_ > 55 mmHg for ≥ 10 min or an increase in TcPCO_2_ ≥ 10 mmHg above awake supine value to a value exceeding 50 mmHg for ≥ 10 min [18]. Clinically relevant threshold values may differ according to 1/the method and device used, 2/the etiology of chronic respiratory failure, 3/the goal of TcPCO_2_ recording (i.e. to decide when NIV should be initiated or to monitor NIV efficacy) and 4/PCO_2_ levels when NIV is started. For example, prognosis is improved in COPD if NIV effectively reduces PaCO_2_ by more than 20% [38]. The thresholds used may also depend on the type of capnograph as bias between arterial and transcutaneous values changes according to the device used [39]. The device used in our study slightly overestimated PaCO_2_. The maximal bias published with this device was 5.6 ± 3 mmHg [40]. We therefore considered residual nocturnal hypoventilation as significant when mean TcPCO_2_ was ≥ 50 mmHg [41].

The clinical contribution of nocturnal transcutaneous capnography can be improved by simultaneously recording SpO_2_ [19]. Sampling rate and averaging of SpO_2_ and TcPCO_2_ recordings are different: SpO_2_ can detect short desaturations linked to short ventilatory events while TcPCO_2_ has a longer lag time but is an accurate tool to evaluate overnight trends in ventilation. Hence, both tools are complementary and devices used in clinical practice combine TcPCO_2_ and SpO_2_ sensors. However, capnography does not provide information about the underlying pathophysiological mechanisms. Furthermore, in a quarter of patients with normal TcPCO_2_ and SpO_2_ (*strategy* B), we found significant leaks or abnormal residual respiratory events (ie, flow reduction or patient-ventilator asynchronies). Our study confirms the additional contribution of data from ventilator software for the detection of these events. The accuracy of the ResScan™ system used to assess leaks has been confirmed in a bench model by our group and others [8, 42].

Our results suggest that using more severe thresholds for PaCO_2_ and NPO may compensate their lack of sensitivity. For instance, using a PaCO_2_ threshold value of 42 mmHg could increase the accuracy of ABG for the detection of nocturnal hypoventilation.

Time spent with a SpO_2_ below 90% is the most frequently used parameter to interpret nocturnal pulse oximetry, but threshold values vary considerably between authors and aetiologies. In non-ventilated patients suffering from chronic obstructive pulmonary disease (COPD), Levi Valensi et al. [43] documented a shorter survival in patients spending more than 30% of total sleep time with an SpO_2_ below 90%. More recently, Gonzalez-Bermejo et al. [14] showed that ALS patients under NIV had a better survival if less than 5% of NPO time was spent with an SpO_2_ < 90%. In our study, using a threshold of 5% increased the accuracy of NPO in detecting residual nocturnal hypoventilation.

An analysis combining the signals provided by TcPCO_2_ and data from ventilator software may be an interesting option for monitoring NIV, offering a noninvasive global estimation of NIV efficacy without requiring ABG. Moreover, this approach enables unattended assessment both at the hospital and at home without complex logistics. Failure to retrieve data is rare [44] and instrumental drift of TcPCO_2_ is a minor problem when used by an experienced team [20, 39, 45, 46]. Interpretation of the results is simple and further analysis of detailed raw data provided by ventilator software can help clarify the underlying mechanism implicated in NIV inefficacy. This may allow optimization of ventilator settings limiting PSG to more complex cases. Unfortunately, use of TcPCO_2_ is at present still limited by the cost of the devices.

We acknowledge a few limitations to our study. Firstly, we did not perform full PSG under NIV. Even if PSG allows the evaluation of patient-ventilator interactions and characterization of abnormal respiratory events occurring under NIV [7], the impact of these events on morbidity and related therapeutic end points remains speculative [47]. Furthermore, it does not provide an accurate estimation of alveolar ventilation per se*,* which is the main goal of ventilator assistance. It is also probable that leaks could be underscored by PSG.

Secondly, we excluded 32 patients with nocturnal NIV and oxygen therapy. Supplemental oxygen impacts on SpO_2_ values and reduces the amplitude of desaturations, decreasing the reliability of NPO to assess NIV efficacy. It must be noted that the majority of excluded patients suffered from chronic obstructive pulmonary disease.

Thirdly, NIV is considered beneficial if used more than 4 h per night (for ALS [48]; for COPD [49]; for obesity-hypoventilation syndrome [50]). We also excluded patients using NIV for less than 4 h per night. Poor compliance to NIV may result from discomfort related to leaks or a low perceived benefit of treatment. This could have underestimated the proportion of inadequately ventilated patients even if leaks represent the most frequent abnormality in our study.

Fourthly, we failed to show an impact of NIV efficacy on sleep quality or patient symptoms. Both scores employed for assessing comfort and quality of sleep have been previously used to assess subjective impact of changes in ventilator modes (volume-targeted versus conventional bi-level pressure support) [25]. Our results suggest that subjective assessment does not suffice for the detection of inappropriate ventilation. The poor correlation between residual respiratory events and patients’ perception has been previously reported [9, 10]. Finally, the impact of NIV efficacy on survival could not be assessed due to the heterogeneity of our population consisting of subgroups (OLD, CWD, NMD) with different prognoses. Further investigations are needed to identify which of the selected tools included significantly impacts on patient-related outcomes such as symptoms, health-related quality of life or survival.

In summary, this study shows that combining morning ABG and nocturnal SpO_2_ is not sufficient to accurately assess NIV efficacy. An alternative strategy combining data from ventilator software and TcPCO_2_ performed better for detecting inappropriate NIV without requiring ABG. Models of care for chronically ill patients living at home are evolving with tele-monitoring. TcPCO_2_ and ventilator software data are increasingly available at home. Moreover, their easy interpretation makes it feasible in real life and in a variety of clinical settings. This combination may be very useful in future strategies for long-term NIV monitoring.

## Supplementary Information

**Additional file 1.** Patient’s rating quality of sleep and ventilation assessed by St. Mary’s Hospital Questionnaire and eight visual analogic scales according to aetiological groups and objective efficacy of NIV.

## Data Availability

The datasets generated and analysed during the current study are available from the corresponding author on reasonable request.

## Abbreviations

ALS: Amyotrophic lateral sclerosis
ABG: Arterial blood gases
BMI: Body mass index
CHRF: Chronic hypercapnic respiratory failure
COPD: Chronic obstructive pulmonary disease
CWD: Chest wall diseases
NIV: Noninvasive ventilation
NMD: Neuromuscular disease
OLD: Obstructive lung diseases
PaO_2_: Arterial dioxygen partial pressure
PaCO_2_: Arterial carbon dioxide partial pressure
PSG: Polysomnography
SpO_2_: Transcutaneous pulsed oxygen saturation
TcPCO_2_: Transcutaneous carbon dioxide partial pressure
