# Supplementary material for: Monitoring of noninvasive ventilation: comparative analysis of different strategies
Source: Respir Res. 2020 Dec 10;21:324. doi: 10.1186/s12931-020-01586-8 (PMC7725884; doi:10.1186/s12931-020-01586-8)
Supplement: Supplementary file 1 — Additional file 1. Patient’s rating quality of sleep and ventilation assessed by St. Mary’s Hospital Questionnaire and eight visual analogic scales according to aetiological groups and objective efficacy of NIV. [file 12931_2020_1586_MOESM1_ESM.docx]

|  | **OLD**  **(n=25)** | **CWD**  **(n=29)** | **NMD**  **(n=46)** | **Appropriate NIV (n=29)** | **Inappropriate NIV (n=71)** | **Global population (n=100)** |
| --- | --- | --- | --- | --- | --- | --- |
| **St. Mary’s Hospital sleep questionnaire** | | | | | | |
| Depth of sleep  (scale range: 1-8) | 5 [3-6] | 6 [4-7] | 5 [2.2-5] | 5 [2-6] | 5 [3.5-6] | 5 [3-6] |
| Number of awakening | 3 [1-4] | 2 [1-3]* | 3 [2-5]* | 2 [1-5] | 3 [1-4] | 2.5 [1-4] |
| Duration of sleep  (hours) | 5 [4-7] | 5 [5-7] | 6 [5-7.5] | 5 [5-6.7] | 6 [4-7] | 5 [4-7] |
| Quality of sleep  (scale range: 1-5) | 4 [3-4] | 4 [4-5] | 4 [3-4] | 4 [3-4] | 4 [3-4.2] | 4 [3-4] |
| Morning sleepiness  (scale range: 1-6) | 4 [4-5] | 5 [4-5] | 4 [4-5]* | 4 [4-5] | 4 [4-5] | 4 [4-5] |
| Satisfaction with quality of sleep (scale range: 1-5) | 4 [3-5] | 4 [4-5] | 4 [3-4] | 4 [3-4] | 4 [3-5] | 4 [3-5] |
| Early awakening  (yes/no) | 3/22 | 5/24 | 10/36 | 4/25 | 10/61 | 19/81 |
| Difficulty in falling asleep  (scale range:1-4) | 1 [1-2] | 1 [1-1.5] | 1 [1-2] | 1 [1-2] | 1 [1-2] | 1 [1-2] |
| Time needed to fall asleep  (hours) | 1 [0.5-1.5] | 0.5 [0.2-1] | 1 [0.2-2] | 1 [0.2-1] | 1 [0.2-1] | 1 [0.5-1] |
| **Perceived quality of ventilation** | | | | | | |
| Total score | 71 [64-77] | 73 [69-77] | 70 [63-74] | 71 [61.5-75.5] | 71 [65-76] ^¶^ | 71 [65-75.7] |
| Quality of sleep | 8 [6-10] | 9 [7-10] | 7 [5-9]* | 8 [5-9] | 8 [6-10] | 8 [5-10] |
| Morning headache | 10 [10-10] | 10 [9-10] | 10 [10-10] | 10 [10-10] | 10 [10-10] | 10 [10-10] |
| Noise of ventilator | 10 [8-10] | 10 [9-10] | 9 [8-10] | 10 [8-10] | 10 [8-10] | 10 [8-10] |
| Perception of leaks | 10 [7-10] | 9 [8-10] | 9 [7-10] | 9 [7-10] | 9 [8-10] | 9 [7-10] |
| Too little air delivered | 10 [10-10] | 10 [9-10] | 10 [9-10] | 10 [9-10] | 10 [10-10] ^¶^ | 10 [9.5-10] |
| Too much air delivered | 10 [10-10] | 10 [9-10] | 10 [9-10] | 10 [9-10] | 10 [9-10] | 10 [9-10] |
| Asynchronism perceived | 10 [8-10] | 10 [9-10] | 10 [8-10] | 10 [8-10] | 10 [8-10] | 10 [8-10] |
| Comfort of ventilation | 8 [6-10] | 9 [8-10] | 8[7-10] | 8 [8-10] | 8 [7-10] | 8 [7-10] |

Supplemental data : Patient’s rating quality of sleep and ventilation assessed by St. Mary’s Hospital Questionnaire and eight visual analogic scales according to aetiological groups and objective efficacy of NIV

Data are presented as median [quartiles].

Abbreviations: CWD: chest wall diseases; NIV: noninvasive ventilation; NMD: neuromuscular diseases; OLD: obstructive lung diseases.

Patients are considered as appropriately ventilated when nocturnal pulse oximetry + arterial blood gazes + built-in polygraphy + capnography (referred as *strategy A*) were normal.

* p<0.05 for comparisons to CWD group (Kruskall-Wallis test then Dunn’s post-hoc analysis)

^¶^ p<0.05 for comparisons between patients with appropriate or inappropriate ventilation (Mann-Whitney test)
